# SARS-CoV-2-Related Olfactory Dysfunction: Autopsy Findings, Histopathology, and Evaluation of Viral RNA and ACE2 Expression in Olfactory Bulbs

**DOI:** 10.3390/biomedicines12040830

**Published:** 2024-04-09

**Authors:** Marco Dell’Aquila, Concetta Cafiero, Alessandra Micera, Egidio Stigliano, Maria Pia Ottaiano, Giulio Benincasa, Beniamino Schiavone, Leo Guidobaldi, Luigi Santacroce, Salvatore Pisconti, Vincenzo Arena, Raffaele Palmirotta

**Affiliations:** 1 Anatomic Pathology Unit, Department of Woman and Child Health and Public Health, Fondazione Policlinico Universitario A. Gemelli IRCCS, 00168 Rome, Italy; mzrk07@gmail.com (M.D.); egidio.stigliano@policlinicogemelli.it (E.S.); vincenzo.arena@rm.unicatt.it (V.A.); 2 Pathology Unit, Belcolle Hospital, ASL Viterbo, 01100 Viterbo, Italy; 3 Medical Oncology, SG Moscati Hospital, 74010 Statte, Italy; salvatorepisconti@hotmail.it; 4 Anatomic Pathology Unit, Fabrizio Spaziani Hospital, 03100 Frosinone, Italy; 5 Research and Development Laboratory for Biochemical, Molecular and Cellular Applications in Ophthalmological Science, IRCCS–Fondazione Bietti, 00184 Rome, Italy; 6 Department of Clinical Pathology and Molecular Biology, Pineta Grande Hospital, 81030 Castel Volturno, Italy; mariapia.ottaiano@gmail.com (M.P.O.); giulio.benincasa@pinetagrande.it (G.B.); beniamino.schiavone@pinetagrande.it (B.S.); 7 Cytodiagnostic Unit, Section of Pathology Sandro Pertini Hospital, ASL Rm2, 00157 Rome, Italy; leoguidobaldi@gmail.com; 8 Section of Microbiology and Virology, Interdisciplinary Department of Medicine, School of Medicine, University of Bari “Aldo Moro”, 70124 Bari, Italy; luigi.santacroce@uniba.it; 9 Section of Sciences and Technologies of Laboratory Medicine, Interdisciplinary Department of Medicine, School of Medicine, University of Bari “Aldo Moro”, 70124 Bari, Italy; raffaelepalmirotta@gmail.com

**Keywords:** olfactory bulb, SARS-CoV-2, anosmia, neuropathogenesis, COVID-19

## Abstract

Background: The COVID-19 pandemic has been a health emergency with a significant impact on the world due to its high infectiousness. The disease, primarily identified in the lower respiratory tract, develops with numerous clinical symptoms affecting multiple organs and displays a clinical finding of anosmia. Several authors have investigated the pathogenetic mechanisms of the olfactory disturbances caused by SARS-CoV-2 infection, proposing different hypotheses and showing contradictory results. Since uncertainties remain about possible virus neurotropism and direct damage to the olfactory bulb, we investigated the expression of SARS-CoV-2 as well as ACE2 receptor transcripts in autoptic lung and olfactory bulb tissues, with respect to the histopathological features. Methods: Twenty-five COVID-19 olfactory bulbs and lung tissues were randomly collected from 200 initial autopsies performed during the COVID-19 pandemic. Routine diagnosis was based on clinical and radiological findings and were confirmed with post-mortem swabs. Real-time RT-PCR for SARS-CoV-2 and ACE2 receptor RNA was carried out on autoptic FFPE lung and olfactory bulb tissues. Histological staining was performed on tissue specimens and compared with the molecular data. Results: While real-time RT-PCR for SARS-CoV-2 was positive in 23 out of 25 lung samples, the viral RNA expression was absent in olfactory bulbs. ACE2-receptor RNA was present in all tissues examined, being highly expressed in lung samples than olfactory bulbs. Conclusions: Our finding suggests that COVID-19 anosmia is not only due to neurotropism and the direct action of SARS-CoV-2 entering the olfactory bulb. The mechanism of SARS-CoV-2 neuropathogenesis in the olfactory bulb requires a better elucidation and further research studies to mitigate the olfactory bulb damage associated with virus action.

## 1. Introduction

The first reports of COVID-19 emerged in China in 2019 and spread rapidly around the world, and it currently remains a global health emergency [1]. SARS-CoV-2 was first identified through deep sequencing analysis of respiratory tract samples from infected patients [2,3]. Although the virus has been thoroughly studied, its pathobiology remains incompletely understood and some features of COVID-19 infection remain unclear [4,5]. Clinical autopsy played a fundamental role in the understanding of the clinicopathological basis of the disease, confirming its fundamental role in the comprehension of the disease [6,7,8,9,10]. COVID-19 syndrome is characterized by multisystemic symptoms, primarily including a characteristic acute respiratory distress syndrome with a histopathological picture of diffuse alveolar damage, often associated with multiorgan failure [3,4,11]. In addition, COVID-19-affected patients referred a significant range of neurological symptoms including headache, ischemic strokes, hemorrhagic encephalopathy, cranial polyneuritis, and meningitis. Among these, anosmia was also registered as often occurring as a primary or early symptom even in mild infections [12,13,14,15].

Besides the considerable literature on the topic, central nervous system infection and subsequent neurological symptoms attributable to SARS-CoV-2 remain a poorly defined topic [14,15,16,17]. During the COVID-19 pandemic, several theories have been proposed for SARS-CoV-2-induced anosmia. Among others, the most discussed hypothesis refers to a direct mechanism determined by the neurotropic capacity of the virus and the direct invasion of the central nervous system (CNS), and an indirect one mediated by the inflammatory process and the cytokine storm characteristic of SARS-CoV-2 infection [12,14,17,18].

Based on the mechanism of action of other viruses, it was also hypothesized that SARS-CoV-2 could enter the dendritic cilia of olfactory sensory neurons from the nasal cavity at the level of the olfactory epithelium and localize in the olfactory bulb by axonal transport across the cribriform plate [18,19,20].

However, the histopathological findings of SARS-CoV-2 in the olfactory bulb and brain sections are largely unspecific and characterized by the presence of reactive gliosis and signs of hypoxia, sometimes with a mild inflammatory infiltrate, and without clear histopathological evidence of viral infection [12,19,21,22].

As for the possible localization of the virus in the olfactory bulb (epithelium) and brain tissues, numerous studies were carried out on both animal models and autopsy specimens from COVID-19 patients, providing contradictory results. On the other hand, although neurotropism of SARS-CoV-2 has been demonstrated in experimental models of human brain organoids [23], the presence of SARS-CoV-2 in the human olfactory bulb and brain showed conflicting results in different cohorts of COVID-19 patients, depending merely on the presence [12,15,21,24,25] or absence [16,17,26] of the virus. Regarding the olfactory epithelium, an established finding demonstrated that SARS-CoV-2 virus has a tropism for olfactory sustentacular cells, while at the same time, no tropism for sensor neurons was found [16,17,27].

The ACE2 receptor is known to play a central role in the mechanisms of host cell entry during SARS-CoV-2 infection and COVID-19 progression. This protein is expressed in a variety of tissues such as the respiratory epithelia, cardiovascular system, gastrointestinal tract, genitourinary system, and many other tissue types as reported in the Human Protein Atlas database (https://www.proteinatlas.org/) (accessed on 1 January 2020) [4,28,29,30]. In this context, the expression of the organ-specific ACE2 receptor may help in understanding the potential routes of virus infection and explaining the clinical symptoms and mechanisms of viral susceptibility. 

Therefore, the aim of our study was to investigate the presence of SARS-CoV-2 and ACE2 in olfactory bulbs of patients who died from COVID-19 syndrome and who had a clinical ante-mortem diagnosis of SARS-CoV-2 based on both clinical symptoms and radiological imaging, which was then confirmed with post-mortem swabs. The histopathological features of lung and olfactory bulb tissues of each patient were also evaluated and correlated with SARS-CoV-2 and ACE2 expression.

## 2. Materials and Methods

### 2.1. Biosamples’ Collection, Autopsy, and Ethical Issues

Over 200 clinical autopsies on elderly patients who died from COVID-19 syndrome were carried out at the department of Pathology of Gemelli University Hospital–Catholic University of Sacred Heart, Rome, Italy, in 2020. Most of the patients were elderly patients who contracted the infection in the setting of Healthcare Residences, and subsequently died in the Intensive Care Units of our University Hospital. The diagnosis of COVID-19 was either suspected on a clinical and radiological basis or with the execution of a nasopharyngeal ante-mortem swab.

Autopsies were requested during the COVID-19 pandemic for study reasons since SARS-CoV-2 infection was an emerging disease and in the acute phases of the pandemic [31]. Recommendations to perform autopsies of SARS-CoV-2-infected cadavers were issued by the Istituto Superiore di Sanità (government advisory body; Rome, Italy) [32]. Personal protective equipment was used during the autopsy, including N95 masks, waterproof protective suits, goggles, waterproof aprons, and multiple layers of gloves and lab-coats changed before and after each procedure.

Briefly, post-mortem examinations were performed adhering to a biosafety level of 3 (BLS 3), in an autopsy room equipped with a separate ventilation system. Autopsies were performed according to a modified autopsy approach of the Virchow’s technique: a transdiaphragmatic approach allowing for a shorter time of exposure to the thoracic organs as previously described [33]. This approach allowed for a thorough gross examination of the lungs, including the possibility of lung sample collection for histopathological definition of the disease. Gross examination of the brain was avoided in order to prevent aerosol formation [34]. During autopsies, olfactory bulbs were collected with a trans-sphenoidal approach using a Jamshidi needle by inserting it in the nasal cavity, applying pressure in order to penetrate the cribriform plate, and gaining access to the anterior cranial fossa, thus allowing us to reach for the olfactory bulbs—as it was described by our group [6]. All samples were immediately fixed in formalin (10% solution) for one week and subsequently paraffin-embedded (formalin-fixed paraffin-embedded–FFPE), according to a standard procedure. Therefore, we selected a random sample of 25 patients that underwent autopsy with a clinical diagnosis of COVID-19, who died from respiratory failure from acute respiratory distress syndrome. The clinical evidence of anosmia represented the main inclusion criteria. The olfactory bulbs and lung tissues from leftover material were used for the molecular and histopathological analysis and compared with ante-mortem and post-mortem swab results.

### 2.2. SARS-CoV-2 Detection in Swab

Before death, all patients performed a routine nasopharyngeal swab. After death, COVID-19 diagnosis was subsequently confirmed with a protocol of three post-mortem swabs for SARS-CoV-2 performed during autopsy according to a protocol previously described: a nasopharyngeal swab, a tracheal swab, and a swab for each lung, [6]. Briefly, swabs were collected in viral transport medium (UTM, Copan, Italy) and processed for RNA extraction using NIMBUS automated handling stations (Seegene; Arrow Diagnostics S.r.l., Genova, Italy). SARS-CoV-2 RNA was detected by real-time reverse transcript PCR (RT-PCR) using the Allplex 2019-nCoV Assay (Arrow Diagnostics) targeting the E, N, and RdRP genes of the virus. Amplifications were carried out in a cycler CFX96 Touch Real-Time PCR Detection System (Biorad, Hercules, CA, USA), according to the manufacturer’s instructions. Cycle threshold (Cts) values less than 40 were considered positive for SARS-CoV-2 RNA, while Cts > 40 were considered negative.

### 2.3. Histological Examination of Lung and Olfactory Bulb Sections

FFPE sections (5–10 µm) were stained following the Hematoxylin and Eosin (HE; Bioptica, Milan, Italy) procedure including alcohol dehydration and close-mounting in Permount. Sections were observed with a light transmission direct microscope equipped with a digital camera (Nikon, Tokyo, Japan). The morphological characteristics were defined by two expert pathologists (double-blind fashion) and representative fields were digitally acquired and converted to 8-tiff images for figure assembly (Adobe Photoshop Inc., San Jose, CA, USA).

### 2.4. Molecular Analysis: SARS-CoV-2 Detection and ACE2 Receptor Transcript Expression

Serial formalin-fixed FFPE sections (5–10 µm) were investigated for the expression of the SARS-CoV-2 virus and ACE2 receptor transcripts by real-time RT-PCR. Total RNA was extracted using MagCore Total RNA FFPE One Step Kit Cartridge Code 605 by an automated System MagCore Super extractor (RBC Bioscience Corp., New Taipei City, Taiwan) according to the manufacturer’s instructions. Nucleic acids (total RNA) were quantified using a Qubit Fluorometer (Life Technologies, Carlsbad, CA, USA) and subsequently cryopreserved at −80 °C until further processing.

SARS-CoV-2 detection and ACE2 expression were performed on 50 ng of total RNA for reaction by real-time-RT-PCR using, respectively, the AMPLI-SARS-CoV-2 kit (Dia-Chem srl, Napoli, Italy) targeting the virus nucleocapsid N1/N2 genes and the internal control RP gene, and the probe BIORAD assay (qHsaCEP0051563; Bio-Rad Laboratories, Hercules, CA, USA). Amplifications were carried out with the CFX96 Touch real-time PCR Detection System (Biorad) according to the manufacturer’s instructions. Data processing was performed using CFX Maestro Software version 2.3 and the results were considered positive at Cts < 38 and negative for Cts > 40.

### 2.5. Statistical Analysis

All data are presented as numbers, percentages, and mean ± SD or median. Calculations (descriptive statistics) were made using a computer software package MedCalc 12.7 (MedCalc Software, Ostend, Belgium). The Ct values were obtained at the end of real-time RT-PCR amplification.

## 3. Results

In our hospital, the overall autopsies carried out during the COVID-19 pandemic accounted for about 8% of the total deaths. The main clinical–pathological characteristics of the 25 Caucasian patients included in this study are listed in Table 1. This study included 12 females (48%) and 14 males (52%) (average age 81.54 ± 12.14 yrs, median 82 yrs, range 52–96 yrs). All patients had at least two comorbidities, including chronic kidney disease (n = 17), hypertension (n = 14), type-2 diabetes (n = 13), heart failure (n = 11), atrial fibrillation (n = 10), chronic obstructive pulmonary disease (n = 8), dementia (n = 6), dyslipidemia (n = 7), and chronic heart disease (n = 4). The mean weight was 68 ± 13.64 kgs (median 70 kgs, range 40–90 kgs), while the Body Surface Area (BSA) was found to have a mean of 1.77 ± 0.20 (median 1.79, range 1.31–2.16). The features of each case/tissue are listed in Table 1.

As shown in the table, the nasopharyngeal swabs performed as routine before death indicated SARS-CoV-2 infection except for five cases that were negative or uncertain. Regarding the post-mortem swabs for SARS-CoV-2 performed during the autopsy of two cases and six cases, nasopharyngeal and tracheal swab results were, respectively, negative. Moreover, three cases were also negative for tracheal and lung swabs. No complete negativity for all four post-mortem sampling was observed in any case (Table 1). The lung injury showed a clear pathological picture consistent with SARS-CoV-2 infection, as described in numerous previous studies [35,36]. The main lung pathological findings consisted of diffuse alveolar damage (DAD), in either the exudative phase, the organizing phase, or the fibrotic phase. The patterns of lung injury were either epithelial or vascular with findings of microthrombi or microvascular damage, sometimes with the presence of perivascular lymphocytic cuffing and deposition of hyaline membranes. The pathological findings of lung samples are summarized in Table 2, showing reactive gliosis in all cases observed.

**Figure 1:**
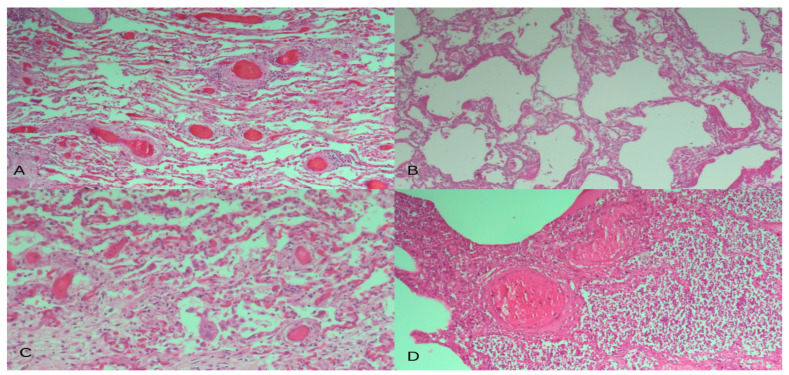
Representative lung parenchyma from HE-stained COVID-19 sections showing (**A**) perivascular chronic lymphocytic inflammation and microthrombi, (**B**) microthrombi and the presence of hyaline membranes, (**C**) the presence of giant multinucleated cells, and (**D**) the exudative phase of diffuse alveolar damage with superimposed bacterial pneumonia. Magnification: ×200.

Since patients were admitted to Intensive Care Unit facilities, superinfected bacterial bronchopneumonia was observed in about 75% of patients (Figure 1). 

**Figure 2:**
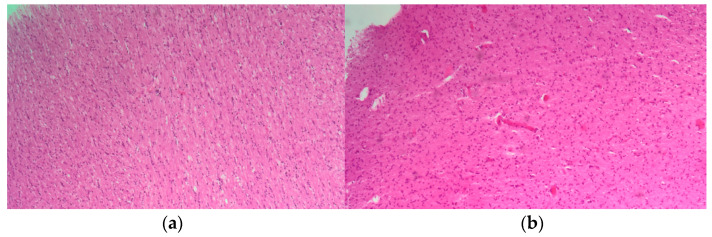
Representative olfactory bulb from HE-stained COVID-19 sections showing (**a**) mild and (**b**) diffuse reactive astrogliosis. Magnification: ×200.

Olfactory bulb samples showed signs of hypoxic damage and non-specific reactive changes of glial cells, with hypertrophy of astrocytes and microglia, and with all characteristics being consistent with a reactive gliosis without microthrombi, intravascular coagulation, or viral inclusion bodies (Figure 2).

Subsequently, lung and olfactory bulb RNAs from FFPE tissues were used to investigate the presence of SARS-CoV-2 virus and the expression of the ACE2 receptors’ transcripts (Table 3).

In FFPE lung tissues, viral RNA was expressed in 23/25 patients, with threshold cycle (Ct) values ranging from 21 to 35 (mean Cts 29.04 ± 3.95; median value 29). By contrast, viral RNA was absent in 21/25 olfactory bulb tissues, and borderline positivity was observed in four cases (merely Cts of 37, 38, and 39 in two cases). ACE2 receptor transcript expression was observed in all FFPE lung tissues with Ct values ranging from 25 to 31 (mean Cts: 27.8 ± 2.01; median value 28). Finally, ACE2 receptor transcript expression was observed in 21 out of 25 olfactory bulbs with Ct values ranging from 26 to 40 (mean Cts: 36.8 ± 4.02; median value: 38 for only 21 samples). The four cases negative to ACE2 receptor amplification were also negative to SARS-CoV-2 expression (Table 3).

## 4. Discussion

The findings of our study confirm and extend previous data on SARS-CoV-2 and ACE2 receptor transcript expression in olfactory bulbs from subjects that died from COVID-19 and referred anosmia. Since SARS-CoV-2 virus was not detected in olfactory bulbs while it was extensively expressed in lung tissues and ante/post-mortem swabs, the hypothesis of a non-direct SARS-CoV-2-mediated anosmia can be proposed and discussed below.

From the outbreak of the SARS-CoV-2 pandemic, olfactory impairment has been one of the earliest and most common symptoms referred by infected subjects, with a prevalence varying widely in the literature, ranging from 5% to 98% depending on the population studied, the geographical area, the diagnostic kits, and the virus variant involved [18,37,38,39]. In this context, a recent study of 616,318 COVID-19 patients in the United States showed that individuals infected with the Alpha variant had a 50% chance of chemosensory disorders. This probability decreased to 44% for the subsequent Delta variant and 17% for the Omicron variant, supporting the hypothesis that patients infected with the newer variants have a significantly lower risk of developing associated chemosensory loss [40].

However, despite numerous studies on this topic, the pathogenesis of COVID-19 anosmia is still not fully understood. Since the onset of the pandemic, several hypotheses have been proposed involving different pathogenetic mechanisms, including putative neurotropism of the virus with invasion of the olfactory bulb, damage to the supporting sustentacular cells of the olfactory epithelium, or altered neuronal function determined by the release of cytokines and subsequent inflammation [14,17,18,19,39,41,42]. Regarding the putative neurotropism of SARS-CoV-2, several reports in the literature indicate the presence of the virus at the level of the olfactory bulb and in different brain areas [12,15,21,24,25,36,43], while other studies do not support the presence of the virus in these areas or in brain regions responsible for the respiratory control [16,17,26].

Recently, in a detailed and comprehensive study of 65 patients who had died a few days after infection with SARS-CoV-2, Khan M et al. used RNAscope and BaseScope in situ hybridization to demonstrate the presence of the virus at the level of the olfactory epithelium in sustentacular cells but not in olfactory sensory neurons [16]. Similarly, the presence of the virus was not found at the level of the olfactory bulb while viral RNA was found in the leptomeningeal layers surrounding the olfactory bulb. Consequently, the authors conclude that SARS-CoV-2 does not have neurotropic capabilities [16]. On the other hand, it has already been shown in animal models that SARS-CoV-2 does not enter the olfactory nerve during anosmia but infects sustentacular cells of the golden Syrian hamster [17,27].

Our results seem to confirm the data from the latter studies and demonstrate the absence of SARS-CoV-2 in olfactory bulbs. The only positives were found in only four samples with extremely high Ct values, so they cannot be considered diagnostic in clinical practice and, in any case, are consistently higher than the average values obtained from lung samples (Table 3). In our case, we cannot exclude that this positivity is due to contamination of the material during the pre-analytical steps, e.g., during microtome cutting. Nevertheless, we believe that any contamination during the autopsy phase was avoided thanks to our minimally invasive trans-ethmoidal approach to the olfactory bulbs [6,33]. Another explanation could be sought in the presence of virions or viral RNAs in infected blood or in blood vessels of the brain, as suggested by some authors who have justified in this way the positivity of the olfactory bulbs found in previous studies [18,38]. Furthermore, it is also feasible that our positive cases may refer to the presence of viral material present in the leptomeningeal layers surrounding the olfactory bulb as previously reported by Khan M et al. [16]. All the olfactory bulbs we examined showed only reactive gliosis, with no clear histopathological signs of viral infection (such as viral inclusions) or clear evidence of viral-induced damage, and without evidence of inflammatory or lymphocytic infiltrate. Reactive gliosis is a non-specific reactive change in glial cells that occurs when there is damage to the central nervous system [44]. As this finding is very non-specific and was found in all our samples, it could be a reactive change to hypoxia or increased inflammatory cytokines following SARS-CoV-2 infection, but it is unlikely to be due to direct viral cytopathic damage.

Since the entry of SARS-CoV-2 is mainly mediated by the ACE2 receptor, we also examined our FFPE samples for the expression levels of this transcript. Our results showed that there was no positivity in the olfactory bulb in four samples, while the remaining samples had higher Ct levels compared to those of the lung. From this, we can conclude that the ACE2 receptor is absent in the olfactory bulb or at least lower than in the lung tissue. In the first phase of the pandemic, Brann D. H. et al. used single-cell RNA sequencing to detect the expression of the ACE2 receptor, which is essential for the entry of SARS-CoV-2 into the cell, in both human and mouse sustentacular cells, horizontal basal cells, and Bowman’s gland cells, elements that function as supporting and stem cells of the olfactory epithelium. Conversely, no transcripts of the ACE2 receptor were found in neuronal elements of the olfactory epithelium and olfactory bulb [20]. At the same time, Klingenstein M. et al. came to similar conclusions using immunohistochemical and immunofluorescence staining and demonstrated in human tissue the distribution of the ACE2 receptor in supporting and stem cells of the olfactory epithelium, but never in any neuronal cell type [45]. In the olfactory bulb, the ACE2 receptor was also not expressed in the axons of olfactory receptor neurons or other neurons, but in limited amounts in the glomerular layer, the mitral cell layer, and in some glial fibrillary acidic protein (GFAP)-positive glia cells [45]. In the same way, by investigating the expression of ACE2 in the brain by analyzing data from publicly available brain transcriptome databases, Chen R et al. suggested a small presence of ACE2 in the olfactory bulb localized, however, exclusively in pericytes and endothelial cells [44]. Later, Ueha R also showed, using immunohistochemical and gene expression methods, that in the olfactory bulb of mice and humans, ACE2-positive cells are found to a limited extent in the glomerular layer and granule cell layer [31].

Therefore, we assume that our data are consistent with the results of these studies and that the minimal ACE2 receptor expression levels we found relate to the relatively small amount of protein distributed in the layers or pericytes and endothelial cells and not in the neuronal cells of the olfactory bulb. In our study, we did not perform an evaluation by immunohistological or immunofluorescence staining methods that would most likely have confirmed the presence of ACE2 in these structures and the absence at the neuronal level as in the previously described studies [20,31,43,44]. Our approach has employed the use of real-time RT-PCR detection, which provides reliable information based on fairly fast, highly reproducible, and easily usable experimental techniques. In addition, the use of ACE2 receptor RNA detection allowed us to compare the results with lung and SARS-COV-2 expression data, using an absolute, linear quantification, and not a binary scale determined by staining intensity that only measures degrees of positivity for immunohistology or immunofluorescence.

Thus, the absence of the SARS-CoV-2 genome and the presence of a low ACE2 receptor expression at the level of the olfactory bulb, as shown by other authors, exclude the neurotropism ability of the virus as a factor responsible for anosmia during COVID-19 infection [16,17,27]. So, to date, although the efforts of various research groups have increased our knowledge on the topic, the pathogenesis of COVID-19 olfactory disorders remains still debated. Arguably, one of the most important potential factors is the inflammatory and immune response that is activated after pathogen recognition, leading to the increased secretion of cytokines and chemokines such as Interleukin-6 (IL-6), Interferon gamma (IFN-γ), Tumor Necrosis Factor-alpha (TNF-α), or Interferon-inducible Protein-10 (IP-10) [14,17,18,45,46]. In this context, the recent study by Finlay J et al. is of great interest, which was carried out on biopsies of the olfactory epithelium of nine patients in whom the loss of the sense of smell persisted for several months after recovery from the disease [47]. The results showed an unusually low proportion of anti-inflammatory M2 macrophages, abnormally high levels of CD207^+^ dendritic cells, and a widespread infiltrate of T cells expressing IFN-γ [46].

Recent transcriptomics studies have also shown very promising results that are likely to elucidate the underlying mechanisms that correlate anosmia with SARS-CoV-2 infection. For example, a study performed on golden Syrian hamsters infected with a low dose of SARS-CoV-2 identifies in the olfactory bulb a complex framework of differentially expressed genes assimilated to Gene Ontology (GO) biological processes related to brain behavior, synaptic plasticity, myeloid leukocyte-mediated immunity, and antigen processing and presentation [47]. On the other hand, we cannot exclude the possibility that one of the potential causes is the presence of nicotinic acetyl-choline receptors in the olfactory bulb, as extensively studied in animal models, which could possibly be inhibited by some SARS-CoV-2 spike glycoproteins, as we had previously suspected [4,5,48]. Moreover, Cafiero C et al. discussed the possibility of a virus-mediated release of small molecules/peptides or by the microenvironment or even by the local microbiota able to affect the cholinergic system that is known to account for the pathological features of COVID-19 [5,48,49]. The hypothesis was supported by previous studies highlighting the presence of circulating/local toxic products in COVID-19 patients [5], strengthening the hypothesis of them being accountable for the clinical manifestations (neurological, hemorrhagic, and thrombotic) observed in COVID-19 patients [5]. Likewise, COVID-19-associated anosmia [50] might find an explanation in this hypothesis and explain why we did not detect a SARS-CoV-2 sequence in olfactory bulbs. The same concept was also proposed for the ocular system (redness and increasing tearing [51]), that might share with the olfactory system (anosmia [50]) the involvement of cholinergic toxicity [52].

We would like to stress that the sequence analysis was not performed in these patients. Note that the earliest data on SARS-CoV-2 genotyping refer to April 20, 2021 [37]. According to these data, the SARS-CoV-2 virus variants predominantly circulating in Italy were VOC202012/01 (so-called UK variant)—lineage B.1.1.7 (91.6%, range: 77.8–100%); P.1 (so-called Brazilian variant) (4.5%, range: 0–18.3%); B.1.351 (0.1%, range: 0–1.4%); and B.1.525 (0.4%, range: 0–7.4%), with some differences between the regions of Northern, Central, and Southern Italy.

## 5. Conclusions

Taken together, (i.) the results obtained from a series of biological samples from patients collected at the time of the first wave of the COVID-19 pandemic in Italy, (ii.) the discrete number of our cohort, and (iii.) the use of simultaneous quantitative analysis of SARS-CoV-2 virus and ACE2 receptor expression in olfactory bulbs are the main strengths of this study.

In conclusion, our study suggests that the true nature of anosmia in COVID-19 syndrome is more complicated than an olfactory bulb colonization by the SARS-CoV-2 virus and is not simply attributed to its neurotropism. The basis of the severity of SARS-CoV-2-mediated pathology could be due to the compromise of the cholinergic system due to an inactivation of the nicotinic receptor which then leads to the blockage of the entire body of the patient in severe cases starting with anosmia. The recent research advances in this area provide hope for a better definition of the pathogenetic mechanisms of anosmia in SARS-CoV-2 infection, allowing the development of specific therapies to counteract or at least alleviate this undesired and limiting symptom in the daily life of COVID-19 patients.

## Figures and Tables

**Table 1:** Clinicopathological characteristics of the study population and result of the RT-PCR assays performed ante-mortem (nasopharyngeal swab) and post-mortem (nasopharyngeal, tracheal, right and left lung).

Case	Age	Sex	Comorbidities	Ante-Mortem Nasopharyngeal	Post-Mortem Nasopharyngeal	Post-Mortem Tracheal	Post-Mortem Right Lung	Post-Mortem Left Lung
1	93	F	hypertension, dementia	Positive	Negative	positive	positive	positive
2	80	F	dementia, T2D, epilepsy	Positive	Positive	positive	positive	positive
3	84	F	previous STEMI, CKD, COPD	Positive	Positive	negative	negative	negative
4	78	F	T2D, hypertension	Positive	Positive	negative	negative	positive
5	90	F	dementia, hypertension	Inconclusive	Positive	negative	negative	positive
6	80	M	AD, essential thrombocythemia, CHD	Positive	Positive	negative	negative	negative
7	92	F	CKD, T2D	Positive	Positive	negative	negative	negative
8	81	M	AF, dementia, T2D	Positive	Negative	positive	negative	negative
9	75	M	CKD, dialysis, T2D	negative	Positive	positive	positive	positive
10	52	M	CHD, CKD	negative	Positive	positive	positive	positive
11	80	M	CHD, CKD	negative	Positive	positive	positive	positive
12	87	F	hypertension, dyslipidemia, T2D, COPD, CKD, AF, HF	positive	Positive	positive	positive	positive
13	61	M	hypertension, dyslipidemia, T2D, COPD, CKD, AF, HF	positive	Positive	positive	positive	positive
14	93	M	hypertension, dyslipidemia, T2D, COPD, CKD, AF, HF	positive	Positive	positive	positive	positive
15	64	F	CKD, T2D, CKD, HF	positive	Positive	positive	positive	positive
16	62	M	previous myocardial infarction, stent, syncope, head trauma, subarachnoid hemorrhage	negative	Positive	positive	positive	positive
17	96	F	dementia, hypertension, dyslipidemia, T2D, COPD, CKD, AF, HF	positive	Positive	positive	positive	positive
18	94	F	hypertension, dyslipidemia, T2D, COPD, CKD, AF, HF	positive	Positive	positive	positive	positive
19	93	M	hypertension, CKD, AF, HF	positive	Positive	positive	positive	positive
20	82	M	hypertension, dyslipidemia, T2D, COPD, CKD, AF, HF	positive	Positive	positive	positive	positive
21	92	M	hypertension, CKD, AF, HF	positive	Positive	negative	positive	positive
22	73	M	hypertension, dyslipidemia, T2D, COPD, CKD, AF, HF	positive	Positive	positive	positive	positive
23	87	F	dementia, heart disease	positive	Positive	positive	positive	positive
24	88	M	hypertension, HF	positive	Positive	positive	positive	positive
25	65	M	hypertension, CHD	positive	Positive	positive	positive	positive

Age (yrs); M: male; F: female; AD: Alzheimer’s Disease; COPD: chronic obstructive pulmonary disease; CKD: chronic kidney disease; HF: heart failure; CHD: chronic heart disease; T2D: type-2 diabetes; AF: atrial fibrillation.

**Table 2:** Lung and olfactory bulb pathological findings associated with COVID-19 syndrome in this study population.

Case	Lung							
	DAD Phase	Thrombotic Micro-Angiopathy	Perivascular Lymphocytic Cuffs	Hyaline Membranes	Pneumocyte Hyperplasia	Superimposed Pneumonia	Alveolar Hemorrhages	Giant Cells
1	exudative	diffuse	Present	Present	Present	bacterial	Present	present
2	exudative	diffuse	Present	Present	Present	bacterial	Present	absent
3	exudative	diffuse	Present	Present	Focal	bacterial	Absent	absent
4	organizing	diffuse	Diffuse	Focal	Diffuse	negative	Present	present
5	exudative	diffuse	Diffuse	Absent	Focal	negative	Focal	present
6	organizing	diffuse	Focal	Absent	Absent	negative	Absent	absent
7	exudative	diffuse	Present	Present	Present	bacterial	Present	present
8	exudative	diffuse	Present	Present	Focal	bacterial	Absent	present
9	exudative	diffuse	Present	Present	Focal	bacterial	Absent	absent
10	organizing	diffuse	Diffuse	Focal	Diffuse	bacterial	Present	absent
11	organizing	diffuse	Diffuse	Focal	Diffuse	negative	Present	present
12	exudative	diffuse	Diffuse	Absent	Focal	negative	Focal	present
13	exudative	diffuse	Diffuse	Absent	Focal	negative	Focal	present
14	fibrotic	focal	Absent	Focal	Absent	bacterial	Present	absent
15	fibrotic	focal	Absent	Focal	Absent	bacterial	Present	absent
16	fibrotic	focal	Absent	Focal	Absent	bacterial	Present	absent
17	fibrotic	focal	Absent	Diffuse	Absent	bacterial	Present	absent
18	exudative	diffuse	Diffuse	Absent	Focal	negative	Focal	present
19	fibrotic	focal	Absent	Diffuse	Absent	bacterial	Present	absent
20	fibrotic	focal	Focal	Diffuse	Absent	bacterial	Present	absent
21	exudative	focal	Diffuse	Absent	Focal	bacterial	Focal	absent
22	exudative	focal	Diffuse	Absent	Focal	bacterial	Focal	absent
23	fibrotic	focal	Absent	Diffuse	Absent	bacterial	Present	absent
24	fibrotic	focal	Absent	Diffuse	Absent	bacterial	Absent	absent
25	organizing	diffuse	Focal	Focal	Diffuse	bacterial	Present	present

DAD: diffuse alveolar damage.

**Table 3:** SARS-CoV-2 and ACE2 transcript expression results as performed on lung and olfactory bulb FFPE tissues by real-time RT-PCR.

Case	Ante-Mortem Nasopharyngeal Swab ^a^	SARS-CoV-2 Lung	SARS-CoV-2 Olfactory Bulb	ACE2 Lung	ACE2 Olfactory Bulb
1	positive	29	negative	29	39
2	positive	30	negative	26	36
3	positive	33	negative	30	negative
4	positive	31	negative	28	38
5	inconclusive	24	38	25	37
6	positive	Negative	negative	30	39
7	positive	34	negative	31	40
8	positive	29	37	28	38
9	negative	Negative	negative	29	39
10	negative	21	negative	25	40
11	negative	24	negative	24	38
12	positive	33	negative	29	negative
13	positive	24	negative	25	38
14	positive	31	negative	28	40
15	positive	29	negative	29	41
16	negative	31	39	27	27
17	positive	35	negative	26	26
18	positive	24	negative	27	39
19	positive	33	negative	29	38
20	positive	28	negative	30	negative
21	positive	29	negative	31	31
22	positive	27	39	26	36
23	positive	33	negative	29	38
24	positive	27	negative	27	36
25	positive	35	negative	30	negative

The data (Ct values) are compared with the results of a nasopharyngeal swab performed ante-mortem. As a rule, Ct values are the inverse of transcript expression. Cts > 40 were considered negative. (^a^) indicates the result of the routine diagnostic SARS-CoV-2 detection test.

## Data Availability

The results and the data analyzed during the current study are available from the corresponding author on reasonable request.
